# The Emerging Roles of Cancer Stem Cells and Wnt/Beta-Catenin Signaling in Hepatoblastoma

**DOI:** 10.3390/cancers11101406

**Published:** 2019-09-20

**Authors:** Nirmala Mavila, Jyothi Thundimadathil

**Affiliations:** 1 Department of Medicine, Division of Digestive and Liver Diseases, Cedars-Sinai Medical Center, Los Angeles, CA 90048, USA; 2 Department of Biomedical Sciences, Division of Applied Cell Biology and Physiology, Cedars-Sinai Medical Center, Los Angeles, CA 90048, USA; 3 Division of Research and Development, Bachem Americas, Inc., Torrance, CA 90505, USA; jtmnair@yahoo.com

**Keywords:** hepatoblastoma, cancer stem cells, pediatric liver cancer, Wnt signaling, beta-catenin

## Abstract

Hepatoblastoma (HB) is the most common form of primary liver malignancy found in pediatric populations. HB is considered to be clonal and arises from hepatoblasts, or embryonic liver progenitor cells. These less differentiated tumor-initiating progenitor cells, or cancer stem cells (CSCs), may contribute to tumor recurrence and resistance to therapies, and have high metastatic abilities. Phenotypic heterogeneity, undesired genetic and epigenetic alterations, and dysregulated signaling pathways provide CSCs with a survival advantage over current therapies. The molecular and cellular basis of HB and the mechanism of CSC induction are not fully understood. The Wnt/beta-catenin pathway is one of the major developmental pathways and is believed to play an important role in the pathogenesis of HB and CSC formation. This review summarizes the cellular and molecular characteristics of HB with a specific emphasis on CSCs and Wnt/beta-catenin signaling.

## 1. Introduction

Hepatoblastoma (HB) is the primary liver cancer found in children generally under three years of age [1]. The term “hepatoblastoma” suggests that these tumors may originate from hepatoblasts, or embryonic liver progenitor cells. HB subtypes are identified using histological subtyping based on the level of cell differentiation. HB tumor aggressiveness is often associated with the stem cell phenotype. Therefore, identifying the mechanisms by which normal stem/progenitor cells are transformed into cancer stem cells is critical for elucidating the molecular and cellular basis of HB. Though a rare type of cancer, the annual incidence of HB has increased in recent years, accounting for nearly 80% of all malignant liver tumors in childhood and comprising about 1% of all pediatric cancers [2,3]. Although the cause of HB is unknown, there are several risk factors associated with its occurrence. These include Beckwith–Wiedemann syndrome, familial adenomatous polyposis, premature birth, low birth weight, conception after infertility treatment, maternal smoking, and a higher maternal pre-pregnancy body mass index (BMI of 25–29) [4,5,6]. Children who are exposed to hepatitis B infection at an early age or suffer from a chronic cholestatic liver disease, such as biliary atresia [7], are also at a higher risk of developing HB. Treatment options vary depending on factors such as tumor stage and metastatic nature. A complete surgical resection and chemotherapy constitute the only primary therapy for HB. Cisplatin-based chemotherapy has become an effective therapy for patients with unresectable tumors and has increased the survival rate of HB patients [8]. Despite the clinical advancements and the high survival rate, liver transplantation is the only treatment recommended in cases of advanced hepatoblastoma in close proximity to vascular structures [1]. Although chemotherapeutics have greatly increased the survival rate, detailed studies are still needed to optimize therapies for patients at high risk of distantly metastasizing and recurrent HB tumors [2,8,9,10].

## 2. Molecular Signatures Associated with HB

HB and HCC (hepatocellular carcinoma) are common liver malignancies that affect different age groups. Even though there are similarities in their tumorigenic characteristics, recent studies have shown that HB differs from HCC not only in its origin, but also in various molecular signatures associated with each malignancy [11]. Global gene expression analysis has demonstrated that genes such as insulin-like growth factor 2 (*IGF-2*), delta-like 1 homolog (*DLK1*), transforming growth factor beta 1 (*TGF beta-1*), mitogen-inducible gene 6 (*MIG-6*), and non-coding RNA *MALAT1* are induced in HB but not in HCC [11]. On the other hand, interferon gamma-inducible protein 27, galectin, ubiquitin 2, and alpha-1 androgen receptor are downregulated in HB but not in HCC [11]. The FXR (farnesoid X receptor)–gankyrin signaling axis is another signaling pathway found to be activated in HB, along with a reduction of tumor-suppressing RNA-binding protein CUGBP1 [12].

**Figure 1:**
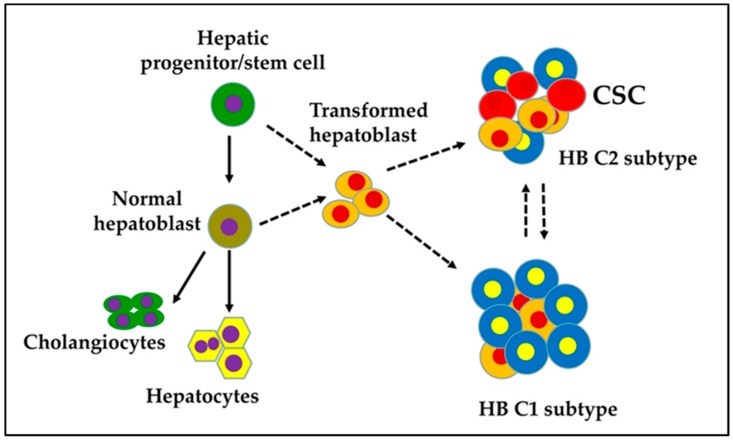
Proposed cellular origin of cancer stem cells (CSC) in a hepatoblastoma (HB).

The presence of various fetal and embryonic liver progenitor markers strengthens the hypothesis that HB originates from transformed hepatoblasts. HB tumors are categorized into two distinct subtypes: C1 and C2 [13]. The C1 subtype recapitulates fetal liver histological features and expresses markers of differentiated hepatocytes. The C2 subtype resembles earlier stages of liver development with an embryonal histology and is characterized by the expression of markers of hepatic progenitor/stem cells (Figure 1). Sequencing studies have shown that HB is one of the pediatric cancers with the lowest rates of somatic mutations. The *CTNNB1* gene, which encodes beta-catenin, is the most frequently mutating gene in exon 3 in HB; consequently, an adenomatous polyposis coli (*APC*) germline mutation in HB has been associated with a familial adenomatous polyposis history. Genomic analysis has revealed a *CTNNB1* mutation rate of ≈68% in HB [14,15,16,17,18,19,20,21,22]. This indicates that Wnt/beta-catenin signaling plays a pivotal role in the malignant transformation of hepatoblasts and HB pathogenesis.

Overexpression of genes, such as *AFP*, *TACSTD1*, *DLG7*, *CDC2*, *BUB1*, *AURKB*, *IGF2*, *DLK1*, *PEG3*, *PEG10*, *BEX1*, *MEG3*, *NDN*, *BIRC5*, *NPM1*, and *HDAC2*, and the activation of Myc signaling have been associated with the HB C2 subtype, which has embryonal characteristics and is enriched with CSCs [13]. On the other hand, C1 subtype HB tumors are characterized by the overexpression of genes such as *GLUL*, *RHBG*, *CYP2E1*, and *CYP1A1*. It is also important to note that different sets of Wnt/beta-catenin-targeted genes are overexpressed in the two HB subtypes [13]. The HB subtypes are also distinguished by the differential expression of metabolic genes such as glucose transporter 3 (GLUT3), lactate dehydrogenase B (LDHB), and glucose-6-phosphate (G6PC), demonstrating distinct glycolytic events in these tumors [23]. Another gene affected by recurrent mutations in HB cases is *NFE2L2* (also known as *NRF2*), which encodes a transcription factor involved in the antioxidant response pathway [14]. *NRF2* mutations are correlated with the activation of nicotinamide adenine dinucleotide phosphate dehydrogenase quinone 1 (NQO1), which has high expression levels in HB and has been found to be associated with invasive and metastatic C2 subtype HB tumors [14]. Comerford et al. developed an interesting mouse model of HB by overexpressing Myc and mutant beta-catenin, wherein mice preferentially developed HB in an NRF2-dependent mechanism [24]. This mouse model mimics molecular characteristics of human HB and demonstrates the synergistical functional role of Myc and beta-catenin, along with an important role of antioxidant pathways, in HB development.

Additionally, cytoplasmic activation/proliferation-associated protein-2 (CAPRIN2), a known oncogene, and tumor suppressors, such as speckle-type POZ protein (SPOP), olfactory receptor-511 (OR5I1), and cell division cycle 20B (CDC20B), have been found to influence HB cell growth. CAPRIN2 is known to activate Wnt signaling via low-density lipoprotein receptor-related protein (LRP) 5/6 phosphorylation [25], and gain-of-function mutations in CAPRIN2 may enhance Wnt/beta-catenin signaling [26]. This study also reported novel mutations affecting components of ubiquitin ligase complexes, including SPOP, (kelch-like 22 KLHL22), transient receptor potential cation channel subfamily C member 4 associated protein (TRPC4AP), and ring finger protein 169 (RNF169) [26]. Studies have found that dephosphorylation of tumor suppressor protein CCAAT/enhancer-binding protein alpha (C/EBP alpha) creates pre-neoplastic foci with CSCs that gives rise to HCC and aggressive forms of HB [27]. These biomarkers can complement existing histopathological methodologies and may aid in a clinical diagnosis that requires distinguishing between aggressive forms of HB.

## 3. Cellular Origin of HB: Transformed Hepatoblasts

HB differs in its developmental origin from HCC in adults. HB occurs in the very early stages of life, while HCC frequently develops on a background of chronic liver diseases. Studies have demonstrated that HB tumors consist of heterogenous populations of stem/progenitor cells [10,13,28]. Therefore, HB is believed to originate from hepatoblasts that undergo undesired genetic/epigenetic aberrations during development (Figure 1). Stemness provides cells with a strong driving force for uncontrolled growth and survival, which may also be associated with metastasis, drug resistance, and tumor recurrence. CSCs possess a high level of plasticity and might undergo undesired genetic, phenotypic, and epigenetic changes in response to the tumor microenvironment, which increase their resistance to therapies and their ability to metastasize to other organs. Because of the phenotypic/genetic heterogeneity, there is no common strategy for targeting CSCs. Hence, CSCs represent the critical subset of cells within the tumor mass in perpetuating the tumor and need to be targeted effectively to prevent HB recurrence and metastasis [2].

CSCs express numerous progenitor/stem cell-specific genes and share many characteristics with normal stem cells. HB tumors have been found to express progenitor markers, such as clusters of differentiation 133 (CD133), EpCAM (epithelial cell adhesion molecule), CD44, CD24, CD90, oval cell antigen-6 (OV6), and aldehyde dehydrogenase1 (ALDH1) [29,30]. CD133, one of the widely studied stem cell markers, is a transmembrane glycoprotein that is significantly upregulated in human liver cancer, as well as in animal models of liver cancer [30,31,32,33,34]. CD133 is also expressed in hepatoblasts, as well as in cholestasis-induced epithelial–mesenchymal liver progenitor cells [35,36]. The CD133 expression profile has been investigated in several clinical studies. Increased CD133 expression levels in human samples have been correlated with tumor stages and alpha-fetoprotein levels, and are characterized by overall lower survival and prognosis and higher recurrence rates [31,37,38]. The CD133-positive tumor cells have exhibited a significantly higher colony-forming efficiency and proliferation ability in vitro and tumorigenicity in xenograft models [34]. The expression of genes involved in self-renewal pathways is markedly higher in CD133-positive cells, and studies have shown that these cells are more resistant to conventional chemotherapy [31,34]. CD133 has been found to be induced by Wnt/beta-catenin signaling in tumor-initiating cells and hepatoblastoma cells [39]. CD133-positivity has been shown to increase chemoresistance via preferential activation of the Akt/PKB and Bcl-2 survival pathways [40]. A recent study found that PIM (proviral integration site for Moloney murine leukemia) kinase promoted stemness via the induction of CD133 in HB cells. PIM kinase inhibition reduced CD133 expression and arrested the growth of hepatoblastoma tumors with CD133-enriched HB cells [41].

EpCAM is another potential CSC marker in the liver. In a normal liver, EpCAM is expressed in the bile duct cells at low levels. EpCAM is a transmembrane protein induced by Wnt/beta-catenin signaling and expressed in a variety of progenitor and stem cells [42], including hepatoblasts and adult hepatic progenitor cells [43,44,45]. EpCAM-positive CSCs have been found to be more tumorigenic and invasive compared to EpCAM-negative cells. Cleavage of EpCAM by tumor necrosis factor-alpha converting enzyme (TACE) or a disintegrin and metalloproteinase domain- containing protein 17 (ADAM17)) and a gamma-secretase complex containing presenilin 2 (PS-2) result in the release of the EpEX domain into the extracellular space and intracellular EpICD domain into the cytoplasm. EpICD directly interacts with beta-catenin and lymphoid enhancer factor (LEF) [46]. The overexpression of EpCAM in hepatic malignancies is associated with a poor survival rate [47]. Due to its high incidence in tumors, EpCAM has been one of the CSC targets in clinical studies, and specific anti-EpCAM antibodies have been tested in cancer patients [48].

CD90 and CD44 have also emerged as markers of liver CSCs. Yang et al. found that the expression of CD90 correlated with tumorigenic potential in liver cancer cell lines. The presence of CD90-positive and CD44-positive cells was found to correlate with tumor metastasis. Blockade of CD44 by a neutralizing antibody induced apoptosis of CD90-positive cells and prevented tumor formation in mice [49].

## 4. Epithelial–Mesenchymal Transition in HB

Epithelial–mesenchymal transition (EMT) is a cellular reprograming process whereby epithelial cells undergo transformation into cells with mesenchymal characteristics. In EMT-activated cells, intercellular junction proteins, such as E-cadherin, are downregulated, while mesenchymal-related proteins, such as N-cadherin, fibronectin, and vimentin, are upregulated. When primary tumor cells undergo EMT, polarized cells become highly migratory and are capable of invasion, leading to distant metastatic colonization [50,51]. Several mechanisms have been linked to EMT in HB. Cancer cells are known to acquire stem-like characteristics through EMT [51,52]. Periostin (POSTN)-induced hepatoblastoma cells undergo EMT, mediated by the mitogen activated protein kinase (MAPK)/extracellular signal regulated kinase (ERK) pathway [53]. DNA damage-regulated autophagy modulator 1 (DRAM1) has been reported to be associated with EMT, migration, and invasion in HepG2, an HB cell line [54]. The long non-coding RNA OIP5-AS1-miR-186a-5p-ZEB1 signaling axis has also been found to regulate EMT in HB [55]. Thymosin beta-4, a known G-actin sequestering factor, modulates the dynamic changes in the cytoskeleton, and has been shown to promote EMT in HB cells [56].

## 5. Wnt/Beta-Catenin Signaling in HB

**Figure 2:**
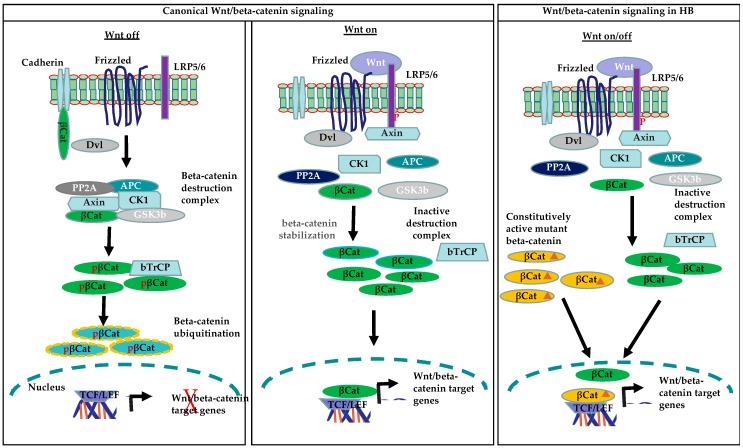
Canonical Wnt/beta-catenin and constitutively active beta-catenin signaling in HB (β-Cat: beta-catenin; pβ-Cat: phosphorylated beta-catenin; β-Cat: mutated beta-catenin)

Accumulating evidence suggests that HB derives from less differentiated cells. Signals that promote self-renewal are one of the key events in the formation of cancer stem cells. Dysregulation of the developmental/self-renewal pathways in normal liver stem/progenitor cells plays a significant role in hepato-carcinogenesis. One of the most important developmental pathways associated with progenitor/stem cells is Wnt/beta-catenin, which is known to play an important role in the activation and expansion of progenitor/stem cells during embryogenesis and liver regeneration, and thus enables hepatic homeostasis [57,58]. Among human cancers tightly linked to abnormal Wnt/beta-catenin signaling, hepatoblastoma (HB) presents the highest rate (up to 90%) of beta-catenin mutations. The activation of the Wnt/beta-catenin signaling pathway is mediated by Wnt proteins, a family of secreted glycoproteins. Wnt ligands bind to cell membrane Frizzled (FZD) receptors and the co-receptor LRP 5/6 to mediate downstream activation of the Wnt/beta-catenin signaling pathway [59,60]. Wnt binding to Frizzled and LRP5/6 triggers the recruitment of the scaffolding protein Disheveled (Dvl), phosphorylation of LRP5/6 and Axin to the plasma membrane. [61]. The recruitment of Axin to the plasma membrane leads to the disruption of the beta-catenin destruction complex, which results in the stabilization and cytoplasmic accumulation of beta-catenin. Beta-catenin is then translocated to the nucleus, where it forms a complex with the T cell factor/lymphoid enhancer factor (TCF/LEF) transcription factors to mediate the expression of Wnt target genes. In the absence of Wnt proteins, the level of beta-catenin is maintained at low levels through its degradation by the destruction complex. This complex consists of the proteins Axin, adenomatous polyposis coli (APC), glycogen synthase kinase 3 beta (GSK3beta), and casein kinase 1 alpha (CK1alpha). Axin, along with other components of the destruction complex, mediates the phosphorylation of beta-catenin at serine 45 by CK1alpha and phosphorylation of serine 33 and 37 and threonine 41 by GSK3beta. Under normal conditions, phosphorylated beta-catenin is recognized by E3 ubiquitin ligase beta-transducin repeat containing E3 ubiquitin protein ligase (beta-TrCP), which triggers the ubiquitination and proteasomal degradation of beta-catenin, thus maintaining its physiological levels in the cells [62]. Deletions of exon 3 of *CTNNB1* or mutations prevent beta-catenin phosphorylation, and thus its degradation leads to an increase in both cytoplasmic and nuclear beta-catenin protein levels, which results in the constitutive activation of the Wnt/beta-catenin pathway (Figure 2).

Global analysis of HB tissues revealed a highly significant role of Wnt/beta-catenin in HB pathogenesis [14]. Oncoprotein Myc, one of the direct targets of the beta-catenin pathway, induced hepatoblastoma-like tumors in mice that resembled the human HB C2 subtype [13]. Genetic aberrations affecting exon 3 of *CTNNB1* in the form of deletions or missense mutations that make beta-catenin stable and constitutively active were found to occur in HB at a rate of nearly 48% [19]. Additionally, Hepatocyte Growth Factor (HGF)/c-Met-mediated beta-catenin phosphorylation, which makes it highly active, was found in nearly 80% of HB tumors [63].

Recurrent mutations in *CTNNB1* and increased expression of the transcription factor *NFE2L2*have been reported in HB [14]. Mutations in other components of the beta-catenin degradation complex, including those affecting APC and Axin have also been reported [19,64,65]. Beta-catenin-mediated expression of hepatic stem/progenitor markers and interplay of Wnt/beta-catenin and Myc signaling have been observed in aggressive HB [13]. A strong nuclear accumulation of both beta-catenin and Yes Associated Protein (YAP) has also been observed in HB tumor samples [66]. YAP is a transcription factor and an important component of the Hippo signaling pathway [67]. Constitutively active beta-catenin and YAP in hepatocytes led to the development of HB in experimental models, suggesting a functional crosstalk between beta-catenin and YAP in the pathogenesis of HB. Interestingly, a beta-catenin–YAP functional interaction was observed specifically in HB and not in HCC or intrahepatic cholangiocarcinoma samples [66]. YAP is activated in approximately 80% of human HB samples, as determined by its nuclear localization [66]. Valanejad et al. showed that the transcriptional regulator poly (ADP-ribose) polymerase 1 (PARP1) binds and activates aggressive liver cancer domains (ALCDs) in HB, which results in the induction of oncogenes, including beta-catenin [68]. Recently, Matsumoto et al. identified growth regulation by estrogen in breast cancer 1 (GREB1) as a novel downstream target of the Wnt/beta-catenin pathway that promoted HB cell growth, both in vitro and in vivo, by suppressing TGF beta signaling [69]. Even though other gene mutations are known to occur in HB, recent studies have demonstrated that genetic defects and aberrant activation of Wnt/beta-catenin signaling components play a significant role in the pathogenesis of HB. These defects can potentially lead to malignant transformation of normal hepatoblasts and eventually development of HB [19,64,70,71,72].

## 6. MicroRNA Regulation of Beta-Catenin Signaling in HB

MicroRNAs (miRNAs) are a family of small, endogenous, non-coding RNAs, approximately 19–25 nucleotides in length. MicroRNAs regulate key signaling pathways in the pathogenesis of hepatoblastoma and are known to regulate CSCs [73,74]. HB tissues have a high expression of miR-371-3 and decreased levels of miR-100/let-7a-2/miR-125b-1. Both of these miR clusters are differentially regulated by Myc, one of the direct targets of beta-catenin signaling [75]. MicroRNAs, such as let-7i-3p, miR-449b-3p, miR-624-5p, and miR-885-5p, downregulate Wnt signaling activity and inhibit HB cell growth. MicroRNAs miR-1246, miR-452, miR-217, miR-612, miR-214, miR-200a, and let7b have also been found to regulate Wnt signaling [76]. Let-7b suppresses the proliferation, invasion and migration of CSCs by inhibiting Wnt/beta-catenin signaling via downregulation of Frizzled 4 [77,78]. MicroRNA miR-1246 promotes the activation of Wnt/beta-catenin in CSCs by cooperating with Octamer 4, an upstream regulator of miR-1246 [79]. The miR-217-DKK1 axis promotes the stemness of cancer cells, resulting in the constitutive activation of Wnt signaling [80], whereas miR-612 suppresses the stemness of liver cancer cells through Wnt/beta-catenin signaling [81]. Ma et al. found that miR-130b facilitates the growth and self-renewal of CD133-positive liver tumor-initiating cells by targeting TP53INP1. Antagonizing miR-130b in CD133-positive tumor-initiating cells significantly increases sensitivity to chemotherapeutic agents and attenuates tumorigenicity in vivo [31]. The miR-200a is known to regulate epithelial–mesenchymal and stem-like transitions via zinc finger E-box binding homeobox 2 (ZEB2) and beta-catenin signaling, respectively [82].

## 7. Therapeutic Targeting of Cancer Stem Cells

**Figure 3:**
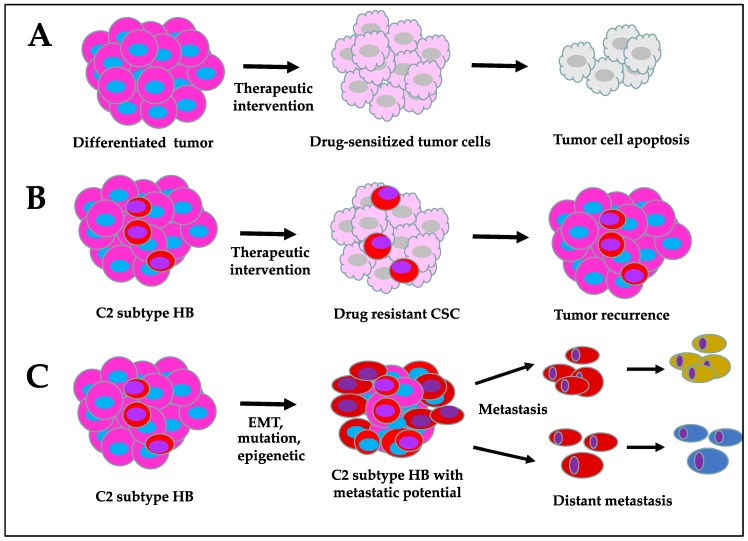
Characteristics of cancer stem cell-enriched hepatoblastoma compared to differentiated tumor: (**A**) drug-sensitive differentiated tumor cells, (**B**) drug resistant CSC in HB, and (**C**) CSC-enriched metastatic HB.

Targeting CSCs is challenging due to their high degree of heterogeneity and the intrinsic survival mechanisms that they develop over time to bypass apoptotic signals. At present, conventional therapies, such as chemotherapy, radiotherapy, and immunotherapy, can effectively suppress the growth of differentiated tumor cells; however, they potentially lag behind in inhibiting CSC proliferation (Figure 3). Although more advanced treatment options have become available in recent years, CSC resistance to therapies and cancer recurrence remain major challenges. CSCs develop therapeutic resistance via cellular reprogramming processes, such as EMT, altered drug tolerating/metabolizing ability, and DNA repair mechanisms, as well as their ability to resist apoptosis [83,84]. The accumulation of epigenetic/mutational changes and the influence by the tumor microenvironment signals may also contribute to chemoresistance. The surface markers of CSCs are generally shared by somatic progenitor cells. High expression of these markers has been observed in human HB tumors. The identification of differentially expressed CSC-specific surface markers, along with aberrantly expressed signaling targets and metabolic alterations, will hopefully aid in distinguishing CSCs from somatic progenitor cells, which may be exploited for selective CSC-targeted therapies. Targeting ATP-binding cassette (ABC) transporters, preventing CSC self-renewal and survival, transforming the more aggressive HB C2 subtype into C1 by inducing cellular differentiation, and developing CSC surface marker-targeting drug delivery approaches are some of the key areas of importance. To achieve these goals, it is necessary to have an in-depth knowledge of the biological characteristics of these cells with respect to their cellular origin, mechanism of propagation, and genetic and molecular defects that distinguish them from normal progenitor cells.

Several basic and clinical studies have demonstrated the critical role for Wnt/beta-catenin signaling in HB. The greatest challenge in targeting Wnt/beta-catenin signaling is its complexity, as it consists of several components regulated by protein post-translational modifications at various levels, as well as extensive signaling crosstalk with other cell-signaling pathways. Targeting nuclear beta-catenin and/or its nuclear regulators responsible for the transcription of downstream Wnt/beta-catenin genes could be a plausible approach to target CSCs. Beta-catenin interaction with cyclic adenosine 3′,5′-monophosphate response element-binding protein (CBP) promotes stem cell renewal, while interaction with P300 enhances progenitor cell differentiation. Therefore, targeting the beta-catenin-CBP interaction is a promising strategy, and small-molecule drugs, such as ICG-001, have been found to effectively target cancer stem cells [39,85,86,87,88]. Inhibition of the beta-catenin–CBP interaction with ICG-001 targets CSCs but not normal cells, and has been reported to arrest cancer growth, both in vitro and in vivo [85,89]. A second-generation ICG-001 drug (PRI-724) is now in clinical trial (NCT01302405). Small-molecule drug FH535 prevents both Wnt and peroxisome proliferator-activated receptor (PPAR)-mediated signaling by suppressing the recruitment of beta-catenin co-activators to target gene promoters and has been shown to be active in different tumor cell lines [90]. PKF115-584, PKF118-310, and CGP049090 are inhibitors of the TCF/beta-catenin protein complex, binding to DNA target sequences [91]. They have been shown to induce apoptosis in vitro and suppress liver tumor growth in vivo regardless of the mutation status of *CTNNB1* [92]. Activation of mammalian target of rapamycin complex 1 (mTORC1) has been reported in human HB cells and in a murine HB model driven by beta-catenin and YAP. Rapamycin, an inhibitor of mTORC1, has been found to significantly arrest HB growth in vivo by inhibiting mTORC1 activation, reducing proliferation, and altering the histology of HB from C2 to the more differentiated C1 subtype [93]. Based on these basic and clinical data, the Wnt/beta-catenin pathway and its interacting partners are some of the promising therapeutic targets that can be used to manage HB recurrence and metastasis.

## 8. Conclusions

Even though several cancer therapeutic approaches are available at present, tumor recurrence is a growing challenge in modern medicine. Recent evidences strongly suggest that CSCs that resist current therapies are the basis of cancer recurrence. Better understanding of the ways in which they originate from normal progenitor/stem or hepatoblast cells, their intrinsic self-renewal and survival mechanisms, and differential drug-metabolizing abilities compared to normal and differentiated cancer cells is critical for developing CSC-targeting therapies.
